# Congenital Heart Disease: An Immunological Perspective

**DOI:** 10.3389/fcvm.2021.701375

**Published:** 2021-08-09

**Authors:** Kavya L. Singampalli, Elysa Jui, Kevin Shani, Yao Ning, Jennifer P. Connell, Ravi K. Birla, Paul L. Bollyky, Christopher A. Caldarone, Sundeep G. Keswani, Kathryn Jane Grande-Allen

**Affiliations:** ^1^Department of Bioengineering, Rice University, Houston, TX, United States; ^2^Medical Scientist Training Program, Baylor College of Medicine, Houston, TX, United States; ^3^Laboratory for Regenerative Tissue Repair, Division of Pediatric Surgery, Department of Surgery, Baylor College of Medicine and Texas Children's Hospital, Houston, TX, United States; ^4^John A. Paulson School of Engineering and Applied Sciences, Harvard University, Cambridge, MA, United States; ^5^Division of Infectious Diseases, Department of Medicine, Stanford University School of Medicine, Stanford, CA, United States; ^6^Division of Congenital Heart Surgery, Departments of Surgery and Pediatrics, Baylor College of Medicine and Texas Children's Hospital, Houston, TX, United States

**Keywords:** congenital heart disease, immune response, inflammation, primary immunodeficiency, models of CHD

## Abstract

Congenital heart disease (CHD) poses a significant global health and economic burden—despite advances in treating CHD reducing the mortality risk, globally CHD accounts for approximately 300,000 deaths yearly. Children with CHD experience both acute and chronic cardiac complications, and though treatment options have improved, some remain extremely invasive. A challenge in addressing these morbidity and mortality risks is that little is known regarding the cause of many CHDs and current evidence suggests a multifactorial etiology. Some studies implicate an immune contribution to CHD development; however, the role of the immune system is not well-understood. Defining the role of the immune and inflammatory responses in CHD therefore holds promise in elucidating mechanisms underlying these disorders and improving upon current diagnostic and treatment options. In this review, we address the current knowledge coinciding CHDs with immune and inflammatory associations, emphasizing conditions where this understanding would provide clinical benefit, and challenges in studying these mechanisms.

## Introduction

Pediatric heart disease, though often overshadowed by its adult counterpart, is a significant health burden, affecting over 15 million children globally (1). Additionally, congenital malformations are a major source of mortality, accounting for accounting for an annual 300,000 deaths worldwide (2). Congenital heart diseases (CHDs) have been reported in anywhere from 3.7 to 75 per 1,000 live births depending on the population, methods of diagnosis, and severity of the disease (3, 4). These heart defects form in utero due to abnormalities in the formation of the cardiac structures and conduction system (5). In many cases, CHDs alter the blood flow pattern throughout the heart as they generate lower pressure pathways that disrupt typical flow; this can have significant morbidity risks as it affects oxygenation and the systemic/pulmonary volume status, which, in turn, can lead to reactionary inflammation (6, 7).

Although vast improvements have been made in CHD treatment and survival (8), they are not without complications. Even in mild cases, children with CHDs report a lower quality of life, poorer performance in school and an inability to physically keep up with their peers (9). Children with more severe CHDs often require serial invasive heart surgeries or transplants (10). Furthermore, these diseases can cause fatal cardiac complications even throughout adulthood (11) and are estimated to reduce life expectancy by nearly 5 years (12).

Despite these complications, there is little conclusive evidence on the specific cause of many structural CHDs, and collectively they are quite heterogeneous. Current research implicates a combination of genetic, epigenetic, and environmental factors as causative mechanisms underlying CHDs (13). A link to the immune system has not been well-defined; however, there is a clear association, as evidenced by an increased risk of these children contracting and experiencing severe complications from common infections. Clinical studies have shown a reduced cellular immune response to infection and increased pro-inflammatory cytokine levels among children with structural CHDs (14–16), which indicates that the immune system may be a dynamic partner in the development of complications from CHDs. Furthermore, immune cells, such as macrophages, have been shown to play a critical role in cardiac development (17). Given how changes in the immune response can be seen in children with CHDs, a potentially fruitful avenue to study pediatric heart disease can be through investigating the immune mechanisms at play.

**Figure 1:**
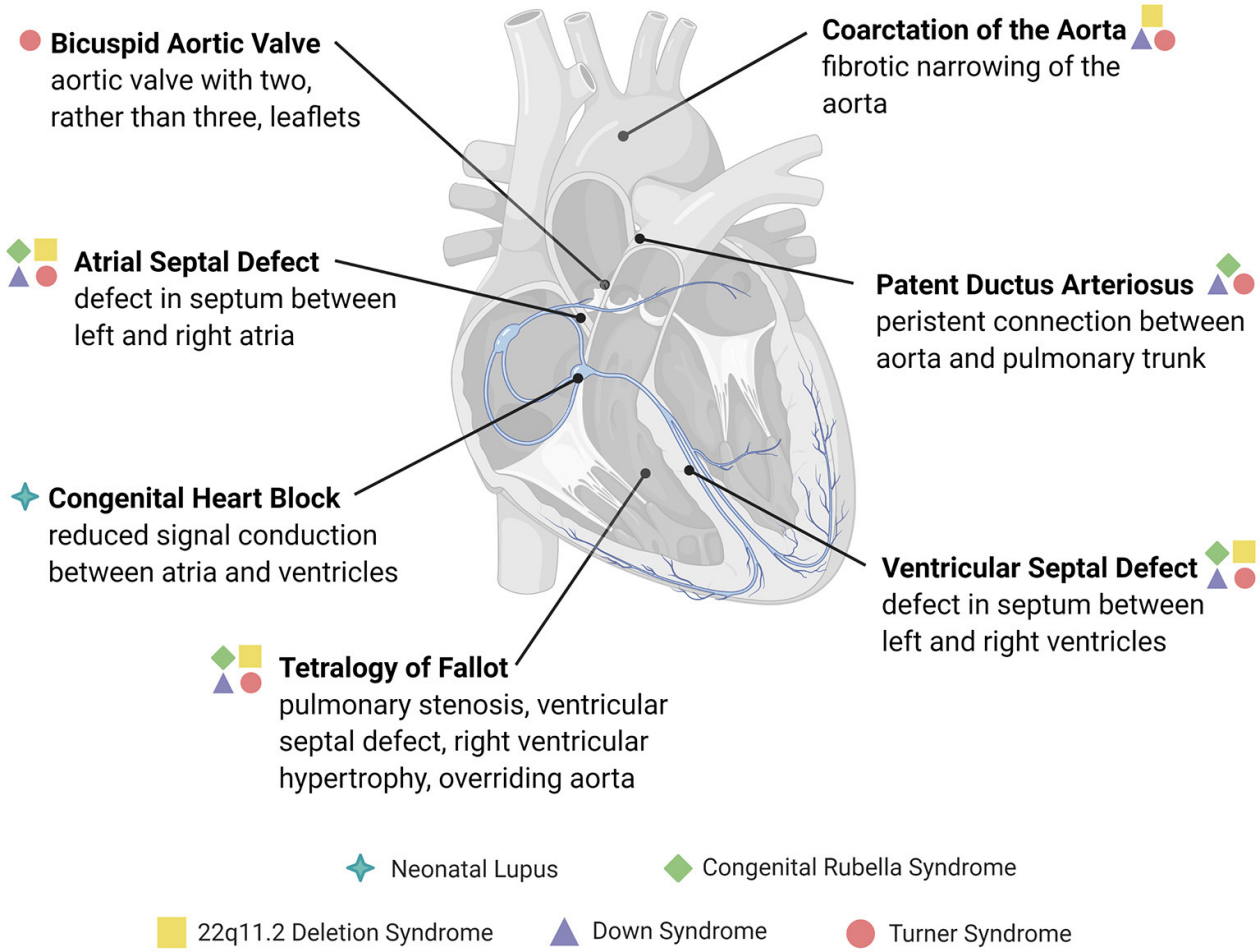
Overview of congenital structural heart defects associated with immune or inflammatory reactions. Congenital heart diseases are conditions present at birth and are mainly comprised of abnormalities in the development of the heart chambers, valves, or the conduction system, or the persistence of congenital structures that typically involute after birth. The heart defects depicted here are examples of those associated with an immune cause or reactive inflammatory changes, many of which are associated with genetic and immune syndromes.

To this end, we discuss the clinical motivation for studying the intersection of the immune system and CHDs, discuss specific CHDs associated with immune or inflammatory complications (Figure 1), and identify challenges in studying the connection between the two. Building on these data, we highlight areas where modulating the immune response may show benefit in preventing the development of disease or its sequelae.

## Clinical Immunologic Implications of CHDs

A motivation for studying the link between immunology and heart disease is that structural CHDs are associated with reduced immune cell counts and maturity. Specifically, children with CHDs have reduced granulocyte activity against bacterial infections (18), T and B lymphocyte levels (14, 19), naïve T-cell production and T-Cell Receptor Excision Circles (TREC) levels (20), IgA and IgG levels, and complement levels, as well as increased suppressor T-cell function (14).

This immune profile becomes clinically relevant, as children with structural CHDs have increased morbidity when exposed to common pathogens, such as respiratory syncytial virus (RSV), and an increased risk of developing bronchopneumonia or other infectious complications. In RSV bronchiolitis, the presence of a CHD can lead to lower oxygen saturations, increased length of hospital stays, likelihood of ICU admission, and up to a 25 × increased risk of mortality (21, 22). Furthermore, in premature infants, CHD is associated with an increased risk of sepsis (23) with up to 35% of late-onset neonatal sepsis presenting in children with Patent Ductus Arteriosus (PDA). These infants are more likely to develop recurrent sepsis, thus requiring longer ventilation times and hospital stays (24). One explanation for the immune complications is the altered anatomy of children with CHDs, specifically the changes in pulmonary circulation. For example, in children with left to right shunts, where increased right-sided flow can cause pulmonary edema or cyanotic heart disease, RSV can be more severe at baseline (25). However, the increased severity of respiratory diseases can also be caused by a shift in immune responses. This is seen in children with CHD who develop bronchopneumonia (BP), who then exhibit an exaggerated increase in both B- and T- cell subsets in addition to the expected increase of CD3^+^ and CD8^+^ cells (26).

The increased susceptibility to infectious complications among children with CHDs may be due in part to the inflammatory response. Although the inflammatory response is elevated at baseline in children with CHDs (6, 15, 27), it is particularly exaggerated in infections, when children present with elevated cytokine and bacterial endotoxin levels (15). Though these inflammatory changes have mainly been observed in small scale clinical studies and there is no consensus on the specific inflammatory profiles, alterations in inflammatory responses have been seen across multiple CHDs. For example, changes in the cytokine profile have been studied as part of a compensatory remodeling mechanism in a variety of structural diseases, including septal defects and shunts. Children born with an atrial septal defect (ASD) undergo cardiac remodeling to compensate for the flow of blood from the left atrium to right atrium. This leads to the presence of markers for mechanical stress, inflammation, and remodeling (28), including tumor necrosis factor (TNF)-α (27). Similar changes in inflammatory cytokines and acute-phase reactants are seen with ventricular septal defects (VSDs) and other left to right shunts (6, 16). In some untreated septal defects or left to right shunts, the pressures in the right heart can become greater than that of the left heart, leading to the reversal of flow, known as Eisenmenger syndrome. This also creates a systemic pro-inflammatory response, with increased C-reactive protein (CRP) and Interferon (IFN)-γ levels (29). Furthermore, in children with coarctation of the aorta, increased inflammatory and apoptotic mediators, including Interleukin (IL)-6, IL-10, TNF-α, and soluble Fas (sFas), may contribute to vascular disease as these children age (30).

Better understanding this inflammatory response can aid in developing prophylactic treatments to prevent complications from CHDs. This has been studied in adult cardiac diseases, where combatting inflammatory markers has been beneficial in therapeutics. For example, heart failure or remodeling after myocardial infarction are associated with a pro-inflammatory cytokine-induced immune response, which, in turn, can cause irreversible fibrosis or systolic dysfunction (31, 32). Specifically, inhibiting molecules such as IL-6 and transforming growth factor (TGF)-β reduces the fibrotic response, making them potential targets for treatment (33, 34). Furthermore, cardiac remodeling in adults is associated with similar cytokine profiles to CHD, including elevated IL-6 and TNF levels (35–37). Though the parallel studies have not been conducted in pediatrics, future large-scale studies can better predict the cytokine profile in CHD, accomplish similar therapeutic success and prevent long-term complications in children.

Since an understanding of the immune and inflammatory contributions to CHD is limited, we have chosen to highlight conditions that establish the range of CHDs with an immune association (Table 1), and the long-term implications of these diseases. In the following sections, we will address examples of CHDs with an immune cause, followed by genetic syndromes involving both CHDs and immune dysregulation. For each, we will discuss the current mechanistic understanding linking the two as well as areas for future research to successfully translate this understanding to clinical practice.

**Table 1:** Immune and inflammatory markers associated with congenital heart disease.

**Disorder**	**Mechanisms**	**References**
Structural CHDs	**↑** Suppressor T cell function	Parikh et al. (18)
	↓ T and B lymphocytes	Khalil et al. (14)
	↓ IgA and IgG	Rhoden et al. (19)
	↓ T cell maturity	Davey et al. (20)
	↓ Granulocyte activity against bacterial infections	
	↓ Complement levels	
Congenital Heart Block	↑ Maternal antibodies against Ro (SSA)-52, Ro-60 or La (SSB)-48	Llanos et al. (38), Rischmuller et al. (39)
	↑ Maternal autoantibodies from Sjogren's syndrome	
Congenital Rubella Syndrome	↑ Rubella antigen	Dhiman et al. (40),
	↑ Cytokine (IL-10, IFN-γ) production	Geyer et al. (41), Lazar et al. (42)
22q11.2 Deletion Syndrome	↓ T and B lymphocytes	Sullivan et al. (43)
	↓ Antibody production	Human et al. (44), Kuo et al. (45), Lambert et al. (46)
Down Syndrome	↑ Toll-Like Receptor 2 signaling	Ram et al. (47)
	↓ T and B lymphocytes	Zampierei et al. (48)
	↓ Effector T cell responsiveness	Farroni et al. (49)
	↓ IgG production	Huggard et al. (50)
Turner Syndrome	↑ IgA production	Stenberg et al. (51)
	↑ Cytokine (IL-6, TGF-β) production	Su et al. (52)
	↓ IgG production	Gawlik et al. (53)
	↓ IgM production	
	↓ T and B lymphocytes	
	↓ CD4:CD8 ratio	
	↓ Cytokine (IL-10) production	

*↑ Means increased levels/activity. ↓ Means decreased levels/activity*.

## Examples of CHDs Associated with Immune Changes

### CHDs With an Immune Etiology

#### Autoimmune-Mediated CHDs: Congenital Heart Block

The prototypical example of autoimmune-mediated CHD is congenital heart block (CHB). CHB is a blockage of the appropriate contractile signaling traveling between the atria and ventricles and is part of a larger constellation of symptoms identified as neonatal lupus, which is correlated to the presence of autoimmune disease in the mother. CHB first can be detected using fetal echocardiography (54); however, at the time of birth, most CHB has progressed to third-degree heart block (55), leading to a 60% pacemaker rate among these children (56, 57). Long-term consequences of CHB include cardiomyopathy, valve disease, endothelial fibroelastosis (58, 59), and a 16% mortality risk (60), all of which necessitate a better understanding of this condition.

A majority of cases are associated with maternal antibodies against Ro (SSA)−52, Ro-60 or La (SSB)−48, as seen in systemic lupus erythematosus (SLE) (61), or autoantibodies associated with Sjogren's syndrome (39). Specifically, the presence of anti-Ro antibodies in maternal serum is associated with a greater risk of progressive CHB and complications, including irregular rhythms, neonatal pacemaker insertion, and spontaneous abortion (62). Newer data show a correlation between antibodies to Ro-52 amino acid residues 1–135 and 200–239, correlated to zinc finger coding regions, and the development of CHB (63).

**Figure 2:**
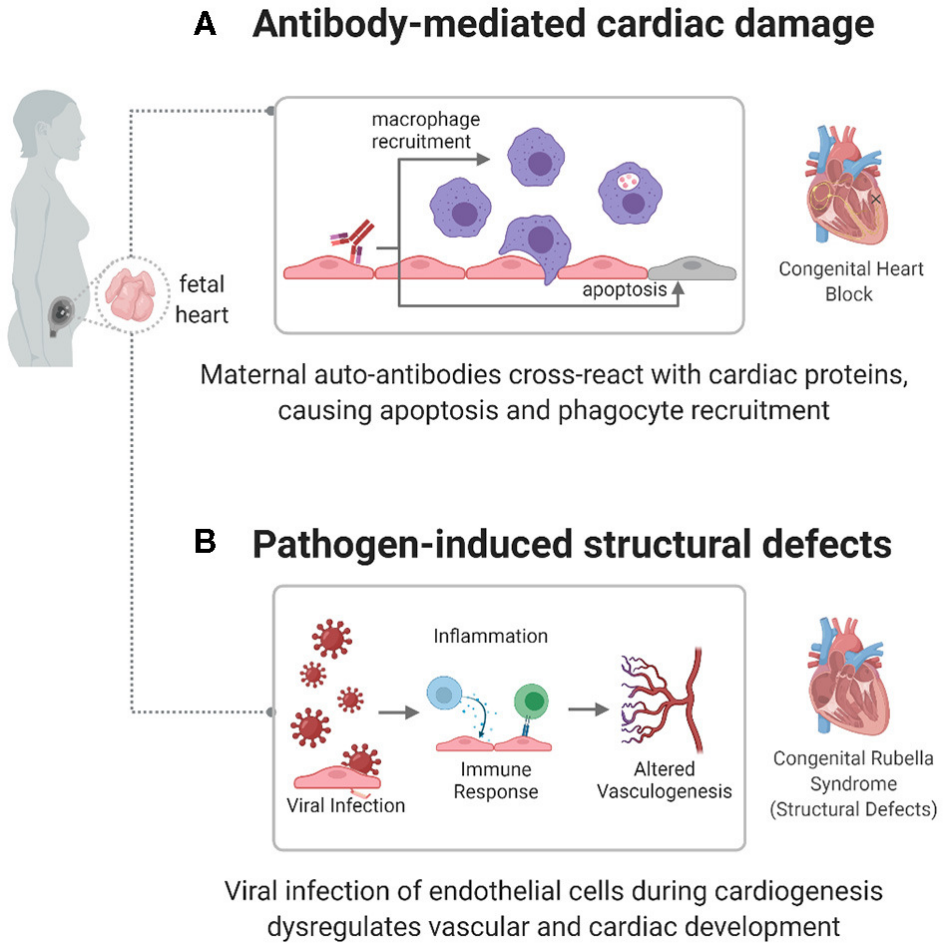
Immune mechanisms causing congenital heart disease. autoimmune and infectious causes have been identified as factors in the development of some CHDs, including congenital heart block (CHB) and congenital rubella syndrome. **(A)** In CHB, maternal autoantibodies target fetal cardiac proteins, causing conductive tissue damage. **(B)** In congenital rubella syndrome, the virus can target endothelial cells during cardiogenesis, preventing normal developmental processes and creating structural defects.

Although an autoimmune component has been established, specific mechanisms connecting autoantibodies to cardiac damage are still being investigated. One mechanism focuses on anti-Ro/anti-La autoantibody targeting of apoptotic cardiac cells (61), as shown in Figure 2A. During apoptosis, Ro and La antigens are translocated to the cell membrane, where they can be targeted by autoantibodies. This leads to opsonization, which attracts macrophages, leading to myofibroblast differentiation, TGF-β release, and fibrosis and calcification of the atrioventricular node and conduction system (64–67). Another mechanism focuses on the cross-reactivity of autoantibodies with L-type calcium channels, present in pacemaker cardiac cells (68), or with laminin (69). However, these pathways need to be better defined to act as potential diagnostic or therapeutic targets in children with CHB.

Due to the combination of challenges in early diagnosis and the irreversible nature of CHB, effective therapies have been difficult to develop. Since the immune origins of CHB have been established, therapies have focused on immunosuppression. However, none of the current treatment options have shown widespread efficacy. Though some research suggests that dexamethasone may prevent the progression of heart block (70, 71), other studies have shown that it is not sufficient to improve outcomes (72). Dexamethasone may show some benefit in combination with a β-adrenergic medication to maintain heart rate (70), but both steroids and β-adrenergic drugs have adverse effects, making their use controversial (73, 74). Intravenous immunoglobulin (IVIG) has also been studied as a potential treatment and is believed to work by outcompeting the anti-Ro and anti-La antibodies for transfer across the placenta (74); unfortunately, IVIG has not been shown to prevent heart block or improve survival (75, 76). Furthermore, hydroxychloroquine, one of the treatments typically used in adult autoimmune disease, has been shown to reduce the development of CHB (77), but there is not sufficient evidence for its efficacy. This lack of adequate treatment options places children with CHB at risk of high pacemaker rates and subsequent complications.

Since CHB is one of the better understood connections between the immune system and congenital disease, it depicts the potential of targeted immunomodulatory therapies for heart disease. A comprehensive understanding of autoimmune mediated pathways can therefore help advance the prevention of maternal autoimmune complications in the fetus and the treatment of CHB in children.

#### Pathogen-Induced CHDs: Congenital Rubella Syndrome

Congenital rubella is a viral infection that postnatally causes rash, lymphadenopathy, fever, and sore throat. Intrauterine infection with the rubella virus, however, can lead to intrauterine growth restriction, cataracts, abnormal brain development, and CHDs, most commonly septal defects, PDA, pulmonary artery stenosis, and Tetralogy of Fallot (ToF) (13, 78, 79).

The mechanism by which the infection causes cardiac defects has not been clearly identified. However, current leading theories include viral damage to vasculature or endothelial cells, affecting the development of tissue early in gestation (Figure 2B) (78). Lazar et al. identified the rubella antigen in cardiac and aortic fibroblasts, placental capillary endothelial cells, and cerebral progenitor cells (42), which match the structures associated with disease phenotypes. Furthermore, the rubella virus has been shown to replicate and persist in endothelial cells *in vitro*, without affecting their shape, structure, or ability to proliferate (80). This infection induces gene expression changes in endothelial cells. Furthermore, induced Pluripotent Stem Cells (iPSCs) infected with rubella and differentiated to an endodermal lineage show an altered expression of endodermal markers and genes related to interferon responsiveness, chromatin remodeling, vasculogenesis, and cell adhesion (81), which induce the recruitment of inflammatory cytokines (IL-10, IFN-γ) and effector lymphocyte responses (40, 41). This connection between altered gene expression and cardiac maldevelopment can help identify specific pathways affected by an immune response in the development of disease.

### Syndromic Associations With Altered Immune Responses and CHDs

**Figure 3:**
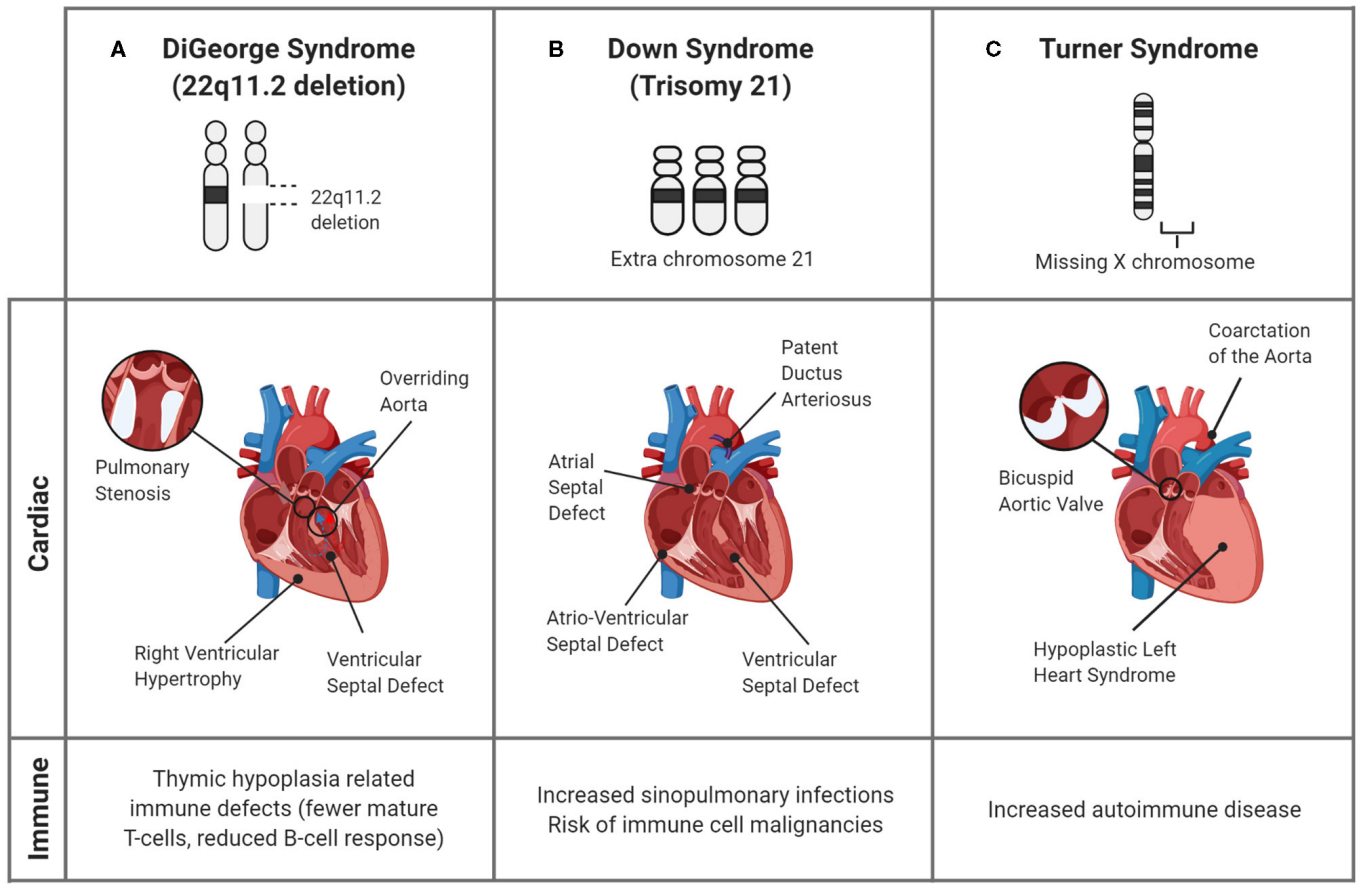
Genetic syndromes associated with CHDs and altered immune responses. Though many genetic syndromes are associated with both immune deficiencies and CHDs, they are not linked directly. **(A)** In DiGeorge Syndrome, the abnormal development of the pharyngeal arches, the precursor structure for both the heart and thymus, leads to mature T-cell deficiency and cardiac defects. **(B)** Down Syndrome is commonly associated with cardiac septal defects and an increased propensity for infections in childhood. Though the associations are unclear, the alterations in immune response are evidenced by the greater prevalence of immune cell malignancies. **(C)** In Turner Syndrome, children can have CHDs, including bicuspid aortic valve and aortic damage, as well as immune complications, such as autoimmune disease. A connection between the cardiac and immune systems is seen in the inflammatory complications of the bicuspid valves, which undergo premature immune-mediated calcification, and induce a systemic pro-inflammatory state.

Many genetic conditions present with both immunologic and cardiac disease manifestations (44). In fact, some CHDs are associated with concomitant genetic immune disorders, collectively termed primary immune deficiencies. Though a comprehensive discussion of these heterogeneous conditions is beyond the scope of this review, there are several relatively common syndromic diseases that present with cardiac and immunologic features as part of a single genetic defect. These conditions, discussed below, include 22q11.2 deletion (DiGeorge) Syndrome, Down Syndrome, and Turner Syndrome (Figure 3).

#### 22q11.2 Deletion Syndrome

A syndrome commonly associated with both immunodeficiency and cardiac disease is 22q11.2 deletion syndrome. In this condition, the third and fourth pharyngeal arches, and their subsequent structures, including the nasopharynx, thyroid, parathyroid, thymus, and cardiac outflow tract can be maldeveloped, leading to palatal defects, hypothyroidism, hypocalcemia, T-cell immunodeficiency, and conotruncal cardiac defects among characteristic facial features (44–46). Since the thymus selects for T-cells that recognize foreign antigens and not self-antigens, thymic hypoplasia causes a T-cell deficiency in up to 75% of these children (43), leading to chronic sinopulmonary infections (82). This reduced thymic T-cell maturation is also evidenced by reduced numbers of TRECs, a byproduct of the selection process (44). The body can attempt to compensate for T-cell lymphopenia through homeostatic expansion, where naive T-cells, often against self-antigens, replicate and mature to restore normal T-cell counts, introducing an avenue for autoimmunity (83).

Additionally, due to the abnormal development of the cardiac outflow tract, up to 80% of children with 22q11.2 deletion syndrome can have congenital heart defects, including VSDs and ToF (84), as depicted in Figure 3A. Studies suggest that a unifying factor between immune and cardiac defects may be altered arterial formation, specifically due to endothelial and neural crest cells. This can lead to improper formation of the cardiac outflow tract and changes in the blood supply feeding the thymus and parathyroid glands (85). Further research to identify the mechanisms that link these deficiencies is crucial to determine deleted genes that are responsible for both immune and cardiac developmental anomalies, which could act as a prognostic or therapeutic target.

#### Down Syndrome

Down Syndrome (DS), also known as trisomy 21, is a condition where children are born with a third copy of chromosome 21 causing a characteristic appearance and clinical comorbidities including developmental delay, a risk of obstructive sleep apnea, gastrointestinal atresias, intellectual disability, and CHD (50). These children are susceptible to frequent and/or prolonged respiratory infections and otitis media, which can be attributed to both altered anatomy and immune changes (47).

Immune complications are focused on the adaptive immune system. Altered myelopoiesis and incomplete fate switching to a memory subtype results in lower numbers of B- and T-cells in up to 90% of children with DS, complicating their responsiveness to vaccines and increasing their risk of autoimmunity (47, 49, 86). Additionally, effector and regulatory T-cell responsiveness and IgG production are reduced in DS (47, 87). Although there is no consensus, potential mechanisms include altered miRNA expression within immune cells, systemic expression of immune-related genes, and increased Toll-Like Receptor (TLR)−2 signaling leading to chronic inflammation (48–50). A potentially fatal complication that supports immune dysregulation in DS is the risk of developing childhood megakaryoblastic or lymphoblastic leukemias. However, these children may have an immune profile that is protective from solid tumors later in life (88). In this case, not only can understanding the immune profile inform cancer diagnosis and treatment in children with DS, but can also provide insight into immune mediators of solid tumors.

Although a direct connection to the immune response has not been elucidated, many of these children have conotruncal cardiac defects, such as atrioventricular septal defects (AVSD) and PDA (Figure 3B), leading to congestive heart failure or valvular insufficiency. Furthermore, these changes expose endothelial cells to altered shear stresses, causing vascular changes including persistent pulmonary hypertension of the newborn (89, 90). The exact mechanism is unknown, however the similarities in defects among children with DS indicate that chromosome 21 encodes genes important for cardiac development (90). The identification of the specific causes would be beneficial since the greatest risk of infant mortality in DS is associated with CHDs (89).

Interestingly, patients with DS have a reduced incidence of coronary artery disease and atherosclerosis, which may be attributed to concomitant increases in protective factors (90). Similar to the immune complications, understanding these mechanisms can allow for the treatment of a major cause of morbidity in the general population.

#### Turner Syndrome

Turner Syndrome (TS) is a female chromosomal disorder that results in a missing or partially missing X chromosome. With an incidence of 1 in 2,000–2,500 births, TS is one of the most common chromosomal defects (53). Though there is often a delay in diagnosis (91), many TS patients have HLHS, bicuspid aortic valves (BAV), aortic stenosis, or coarctation of the aorta (CoA) (92), as illustrated in Figure 3C. As adults, patients also are at risk of left ventricular hypertrophy, coronary artery disease, valve dysfunction (91) and most acutely, the fatal rupture of the thoracic aorta following chronic enlargement (92, 93). The development of these complications may have an inflammatory etiology, as, patients with thoracic aortic aneurysms have an increased number of natural killer, B, and T-cells (94) and inflammatory markers such as TNF-α, IL-1, IL-6, and IL-17 (95).

The risk of autoimmune disease in patients with TS is two times greater than the general female population, suggesting that the immune system plays a clear role in the development of TS complications. Approximately 20–50% of patients with TS are diagnosed with an autoimmune disease (53), with the most common being thyroid disease and diabetes (91). Furthermore, several studies determined that TS patients with an increased risk of autoimmune disorders exhibit a lower percentage of CD4^+^ cells and a lower CD4^+^:CD8^+^ ratio (51–53). Immunological disturbances, such as low levels of IgG, IgM, T- and B-lymphocytes, increased IgA, and pro-inflammatory changes (increased IL-6 and TGF-β, decreased IL-10), have also been associated with TS (51, 91), all of which could serve as predictive factors of complications. It is currently unclear whether the cardiovascular aberrations provoke these immunological deficiencies, or vice versa. However, further studies are warranted given the multitude of evidence linking the two in multiple genetic syndromes.

## Models of the Immune Aspects of CHDs

Despite the advances in studying CHDs, one of the challenges in understanding the immune component is the paucity of *in vitro* or animal models for these diseases. Though syndromic CHDs have been studied in genetic-based animal models and computational models, these do not always lend themselves easily to the study of molecular and immune mechanisms. For example, most mice used to study 22q11.2 deletion syndrome are typically created using single gene mutations (96), which may not encompass the complexity of the disease. Therefore, much of the research in this field is focused on extrapolating data from other diseases or studying clinical manifestations.

*In vitro* models have shown promise in recapitulating mechanisms behind some CHDs. Human induced pluripotent stem cell (hiPSC)-derived cardiomyocytes can be used to model gene regulatory interactions, cell-cell interactions, and tissue interactions contributing to CHD. Specifically, the use of hiPSCs allows for a multitude of benefits such as the direct application to human phenotypes and disease, the ability to study single-cell genomics and epigenetics, scalability to increase detection of low level or transient signaling molecules, the ability to perturb and study developmental interactions that occur at the molecular and cellular levels, and the ability to study tissue organization and interactions using hiPSCs on bioengineered matrices (97). This is especially promising in studying the interactions of immune cells and cardiac cells under specific environmental cues. Furthermore, patient-specific cell lines can be used to investigate multiple pediatric cardiovascular disorders to provide insight to the mechanisms involved and usher in novel personalized therapies for patients.

Computational fluid dynamic models can also provide insight to varied flow patterns and areas of maximum stress in aortic coarctation resulting in patient-specific treatment options (98). These models can predict changes in shear stress, pressure, cardiac energy losses, and PV loops caused by structural differences in these patients (99), which can then be correlated to immune or inflammatory changes seen physiologically with the altered hemodynamics. Computational models are beneficial because they can be individualized to the anatomy of a specific patient. This allows for personalized models of pediatric cardiovascular disorders which can then be compared with immune responses to elucidate trends. Especially since the extent of structural changes in many of these diseases is variable, trends from these computational models can be beneficial in predicting the severity and progression of disease when correlated with clinical data. For example, patient data including inflammatory cytokine levels, CRP, and other immunological profiles can be used as input parameters for the prediction of disease pathogenesis (100, 101). However, these tools still fail to elucidate molecular mechanisms leading to CHDs.

*In vivo* models that have been used to study cardiac development include chicken embryos, zebrafish, and mice. Studying the development of microscopic cardiac structural changes or macroscopic defects in chick embryos involves altering blood flow using drugs or surgical changes, such as banding arteries (102, 103). These models can then be used to study downstream immune and inflammatory changes due to alterations in cardiac flow patterns. Similarly, mouse models of CHDs associated with genetic mutations show a promising avenue for the study of the related immune mechanisms (104). For example, mutations associated with septal defects, such as in NKX2-5 (105) and GATA4 (106), have caused both ASDs and VSDs in mice, whereas mutations in the NOTCH (107) and GATA5 (108) pathways have recapitulated BAVs in mice. These models are beneficial to understand the downstream immune and inflammatory changes of CHDs. However, creating these requires a significant understanding of the genetic causes of CHD, imposes a lack of control on the severity of the defect, and are associated with a long time and high cost for development (104).

Although animal models do not yet exist for many types of structural CHDs, one method of studying their effects is with animal models of the hemodynamic changes associated with CHD. For instance, to model the high cardiac work conditions causing hypertrophy, a pump can infuse isoproterenol, a β-agonist, into pediatric mice (109). Similarly, in diseases where forward flow is impaired, such as coarctation of the aorta or valve stenosis, models of volume or pressure overload can be used as a surrogate. An example would be an aortic coarctation rat model used by Shang et al. (110), which led to increased pressure within the left heart. This allows for the study of inflammatory markers associated with CHDs, including an increase in reactive oxygen species (ROS) and macrophage chemoattractant protein (MCP)−1. Models like this can help in studying the consequences of a particular CHD; however, the specific immune causes of the defects are likely not clearly elucidated from these downstream pathways. This also holds true for larger animal models, such as porcine (111, 112) and ovine (113) models, where the structural heart defect needs to be created by fetal surgery and would therefore not mimic immune mechanisms that cause the initial defects in humans, but could model reactionary inflammatory and immune changes.

The lack of a model to comprehensively study the relationship between the immune system and CHD imposes a challenge to thoroughly understand these conditions. Furthermore, the variability seen in phenotypes of animal models highlights the complexity of the mechanisms underlying these diseases. Despite these limitations, a combination of techniques can be used to study the immune and inflammatory causes of and responses to CHDs. Further refining the models used to study CHDs can expand our current insight into these conditions, which in turn can improve the care of affected children.

## Conclusion

Despite our improved understanding of CHDs and treatment modalities, the role of the immune system in their development or complications is relatively poorly understood. Studies have shown that children with CHDs have altered inflammatory and immune profiles which may contribute to the severe reactions to common infectious agents. However, the specific interaction between the immune system and cardiac defects is not well-understood. Some CHDs, such as defects resulting from congenial rubella or neonatal lupus, have immune mechanisms involved in their development. However, many syndromic associations between CHDs and the immune response have yet to be linked. Additionally, the contribution of the immune system to genetic and environmental factors that cause CHDs has not been well-understood.

A challenge to better defining this relationship is the limits of *in vitro* and *in vivo* models in recapitulating the interactions between the various factors. Much of the current understanding of the immune and inflammatory response in CHD is typically from clinical research and is focused on complications of altered blood flow patterns caused by CHDs. However, by expanding research to a more mechanistic understanding, we can identify specific targets in the development of CHDs or the progression of their complications, thereby expanding our potential to clinically manage both acute and long-term complications of CHDs and reducing the global morbidity and mortality burden imposed on children with these conditions.

## Author Contributions

KG-A conceptualized the article. EJ and KS conducted the literature review, drafted the manuscript, and created the figures. All authors critically edited the article.

## Conflict of Interest

The authors declare that the research was conducted in the absence of any commercial or financial relationships that could be construed as a potential conflict of interest.

## Publisher's Note

All claims expressed in this article are solely those of the authors and do not necessarily represent those of their affiliated organizations, or those of the publisher, the editors and the reviewers. Any product that may be evaluated in this article, or claim that may be made by its manufacturer, is not guaranteed or endorsed by the publisher.
